# Sulfur signaling: from molecules to physiological responses

**DOI:** 10.1093/jxb/erag380

**Published:** 2026-09-29

**Authors:** Stanislav Kopriva, Hideki Takahashi, Markus Wirtz

**Affiliations:** Institute for Plant Sciences, University of Cologne, Cologne, Germany; Cluster of Excellence on Plant Sciences (CEPLAS), Cologne, Germany; Department of Biochemistry and Molecular Biology, Michigan State University, East Lansing, MI, USA; Centre for Organismal Studies (COS) Heidelberg, Heidelberg University, Heidelberg, Germany; Excellence Cluster GreenRobust, Heidelberg, Germany

**Sulfur metabolism plays a central role in determining plant resilience in environments where nutrient availability fluctuates and plants are exposed to both biotic and abiotic stressors. As an essential macronutrient, sulfur is vital for the structural and catalytic integrity of proteins, cofactors, and metabolites that enable plants to withstand drought, salinity, pathogen attack, and oxidative stress. Unlike other mineral nutrients, sulfur is incorporated into a uniquely diverse set of bioactive molecules whose functions extend far beyond features attributed to the primary metabolites. This biochemical versatility makes sulfur not only indispensable for growth but also a key component of the adaptive capacity of plants in the face of dynamic environmental challenges.**

Over the past decades, research in this field has expanded from elucidating the core assimilatory sulfate reduction pathway to identifying its intricate connections with specialized metabolic pathways, uncovering how inorganic sulfate is converted to reduced sulfur metabolites that can be further incorporated into cysteine and a wide array of downstream metabolites. Numerous studies have mapped the metabolic branches that diverge from cysteine and methionine biosynthesis to produce glutathione, phytochelatins, glucosinolates, and other sulfur-containing compounds with critical roles in detoxification, redox buffering, pathogen defense, and environmental stress tolerance. These downstream pathways underscore how sulfur metabolism is tightly connected with cellular homeostasis under stress.

One of the major research topics has been the regulatory architecture that coordinates sulfur metabolism with environmental cues, particularly sulfur limitation. Plants possess sophisticated sensing and signaling mechanisms that adjust sulfate uptake, sulfur metabolic flux, and the allocation of sulfur resources in response to internal demand and external supply. Transcriptional regulators, miRNAs, redox-dependent switches, and metabolite feedback loops coordinate these processes upon plant exposure to sulfur limitation.

Thus, sulfur assimilation not only fuels the production of protective metabolites but also contributes to the generation and modulation of signals that orchestrate adaptive responses. Some endogenous regulators themselves are sulfur-containing molecules, and the biosynthesis of several key signaling compounds depends directly on sulfur metabolism. This Special Issue focuses on recent findings that establish the critical role of sulfur-derived signals in stress adaptation and on how external and internal cues regulate the formation of signals derived from sulfur metabolism.

## Overview of contributions

### Interaction with the environment

Sulfur plays an important role as a nutrient and a signal in interactions of plants with microorganisms. For instance, microorganisms mobilize organic sulfur through sulfatase activity, volatile sulfur production, and sulfoquinovose degradation, making it available for plants. On the other hand, plants shape the composition of microbial communities in the rhizosphere by exuding sulfur-containing defense compounds, such as the phytoalexin camalexin, linking primary nutrient assimilation to immune regulation (Elkatmis *et al.*, 2026). Additionally, beneficial microbes modulate the uptake of sulfur and iron into plants by reprogramming their transcriptional and metabolic networks. Another microbial strategy to improve Fe uptake by plants is siderophore-mediated iron mobilization. How the iron–sulfur metabolism of the plant can be optimized by artificial intelligence (AI)-driven trait-based design of microbial communities is discussed in Garcia-Godos *et al.* (2026).

An imbalance between external iron and sulfur supplies triggers massive nutrient recycling via autophagy, leading to substantial yield losses. How sulfur-related redox regulation of nutrient sensor kinases, such as sucrose non-fermenting 1-related protein kinase 1 (SnRK1) and target of rapamycin (TOR), may contribute to metal-induced autophagy is outlined in Vanbuel *et al.* (2026). Together with a set of nutrient-specific key transcription factors, these kinases are critical for coordinating growth with the fluctuating external supply of macronutrients such as nitrogen, phosphorus, iron, and sulfur. Recent advances in understanding the regulatory networks that maintain homeostasis of these nutrients in *Arabidopsis thaliana* for growth under dynamically fluctuating soil-borne macronutrient supply are summarized in Haque *et al.* (2026). These regulatory networks are influenced by other environmental conditions affecting plant growth, such as temperature and water supply; however, they are less well characterized in crop plants. Lehr and co-workers revealed that the sulfate supply is critical for optimizing water-use efficiency in grapevine (*Vitis vinifera*) and needs to be carefully adjusted to nitrogen availability to prevent metabolic imbalances (Lehr *et al.*, 2026). Also in pea (*Pisum sativum*), sulfur limitation substantially enhanced the detrimental impact of water deficit on plant growth and gene regulation. Among the synergistically up-regulated genes in plants facing low water and sulfur supply, 20 were linked to reactive oxygen species (ROS) responses, with glutathione *S*-transferases being most dramatically induced, probably to control ROS accumulation under this condition (Bonnot *et al.*, 2026).

Phytochelatins, metallothionins, and nicotianamine are critical chelators of metals in the cytosol of plants, facilitating metal tolerability and intracellular metal trafficking. Since all of these compounds are derived from sulfur metabolism, sulfur supply management is also key to optimizing trace element metabolization and metal(loid) detoxification (Wang and Zhao, 2026).

In addition to its specific interactions with other nutrients, sulfur metabolism is of general importance for coping with diverse environmental stresses that lead to ROS accumulation, such as pathogens, drought, and salt stress. Enhanced sulfur metabolism can improve tolerance to such threats by stabilizing the redox status of the cell through glutathione production or by promoting the synthesis of sulfur metabolism-derived defense compounds, such as the osmolyte dimethylsulfoniopropionate (Payet *et al*., 2026). Glucosinolates are another example of sulfur-containing specialized defense compounds designed to broadly protect plants against diverse herbivores. As a consequence, engineering of their biosynthetic pathway in plants is a prime target in agricultural biotechnology. However, due to their nutraceutical value for humans, mass production of specific glucosinolates in microbes is also relevant in white biotechnological approaches (Wajn *et al.*, 2026).

Stress-induced ROS are not only toxic but also serve as critical signals that modulate the glutathione redox milieu, thereby influencing the fate of diverse proteins via glutaredoxin (GRX)-mediated electron transfer. GRXs belong to the thioredoxin superfamily and are organized into four classes in plants. An overview of established and emerging roles of GRXs in plant metabolism is provided in Moseler *et al.* (2026b).

### Transcriptional regulation of and by sulfur metabolism

Diverse stress responses are initiated by substantial remodulation of the transcriptome. The hallmark of the sulfur deficiency response in the reference plant *A. thaliana* is the transcriptional induction of the high-affinity sulfate transporters SULTR1;1 and SULTR1;2 (Takahashi *et al.*, 2011). The groundbreaking discovery was the identification of the EIL family transcription factor, SLIM1 (Sulfur-limitation 1), as a central transcriptional regulator dedicated to controlling the response to sulfur deficiency (Maruyama-Nakashita *et al.*, 2006). SLIM1 also exists in upland and lowland rice varieties to promote root elongation under sulfur limitation (Maruyama *et al.*, 2026), suggesting that this regulatory module is evolutionarily conserved in plants. Besides SULTR1, the gene families encoding the Sulfur Deficiency Induced (SDI) and Response to Low Sulfur (LSU) proteins exist in various plant species and are highly induced during insufficient external sulfur supply. SDI interacts with the MYB28 transcription factor to repress the biosynthetic pathways of the sulfur-containing defense compounds, glucosinolates, thereby prioritizing sulfur use for primary metabolism (Aarabi *et al.*, 2016). Emerging functions of SDIs and LSUs across diverse stress responses are summarized in Sirko *et al.* (2026), suggesting that, in particular, LSUs have broader impacts on plant growth, development, and stress resilience.

Sulfur metabolism-derived signals also control transcriptional responses. DNA methylation is a repressive epigenetic modification that affects gene transcription at a global scale. In all eukaryotes, DNA methylation depends on the availability of the methyl donor *S*-adenosylmethionine (SAM), which is derived from methionine metabolism and is recycled in the Yang cycle (Sauter *et al.*, 2013). Impairing methylthioadenosine nucleosidase, a key enzyme of the Yang cycle, causes developmental abnormalities, global decreases of CG gene body methylation, and massive transcriptome deregulation (Tremblay *et al.*, 2026). How global changes in methionine biosynthesis affect not only SAM-dependent methylation of histones and DNA but also stress responses and plant development is summarized in Amir *et al.* (2026).

### Sulfur signaling: established roles and novel concepts

During diverse abiotic and biotic stresses, sulfur metabolism is critical for the generation of protective molecules that help plants overcome these threats (see ‘Interaction with the environment’). In addition to this classical function, recent work has established that sulfur metabolism also generates stress-specific signaling molecules that modulate transcriptional responses and protein function.

A key signaling molecule derived from sulfur metabolism is the volatile compound hydrogen sulfide. In animals and plants, the best-studied sulfide signaling mechanism is the persulfidation of thiol groups in metabolites or proteins; for example, 887 persulfidated proteins were detected in response to drought stress in plants (Jurado-Flores *et al.*, 2023). In contrast to animals, the majority of sulfide is generated in plant chloroplasts and can be incorporated into cysteine in chloroplasts, the cytosol, and mitochondria (Takahashi *et al.*, 2011). Most probably, the sulfide generated in this anabolic pathway is not the primary source of protein persulfidation and must be tightly regulated in response to cysteine demand for growth and the maintenance of the redox milieu of the cell (Wirtz *et al.*, 2023). In animals and plants, stress-induced protein persulfidation is initiated by the release of sulfide by cysteine-degrading enzymes (Petrovic *et al.*, 2021; Aroca *et al.*, 2026). The diversity of cysteine-degrading enzymes in photosynthetic organisms is broad, and their roles in plant stress physiology are discussed in Moseler *et al.* (2026a). Emerging evidence suggests that protein persulfidation is critical for diverse stress responses, including responses to salinity, high temperature, and drought (Aroca *et al.*, 2026).

A recurring theme is the interconnection between drought stress and sulfur metabolism, which generates metabolites that play significant roles in drought stress signaling and responses (Chan *et al.*, 2013). Like the peptide hormone CLE25, sulfate serves as a xylem-delivered root-to-shoot signal during soil water limitation and must be incorporated into cysteine to trigger stomatal closure (Ernst *et al.*, 2010; Batool *et al.*, 2018). The incorporation of sulfide into cysteine must be specifically facilitated by the chloroplast-localized cysteine synthase complex (pCSC) to trigger the production of the phytohormone abscisic acid (ABA), which controls most aspects of the drought stress response, including stomatal closure. The pCSC in guard cells is also essential for stomatal closure in response to high light and CLE25, thereby defining the pCSC as a hub for integrating diverse stimuli that promote ABA biosynthesis (Sun *et al.*, 2025, 2026a). Emerging functions of the subcellular compartment-specific CSC are outlined in Sun *et al.* (2026b). Independent of the pCSC-induced ABA production, the byproduct of sulfation reactions, 3'-phosphoadenosine 5'-phosphate (PAP), controls the accumulation of high-light-induced and drought-induced genes by inhibiting the 5’ to 3’ exoribonuclease XRN in the nucleus (Estavillo *et al.*, 2011). As PAP steady-state levels are controlled by the organelle-localized enzyme SAL1, the SAL1–PAP signaling axis acts as a chloroplast retrograde signaling pathway that functions in high light and drought signaling. An update on how sulfate metabolism modulates chloroplast-to-nucleus retrograde signaling during oxidative stress by the SAL1–PAP axis and glutathione production is provided in Furbank *et al.* (2026). Remarkably, on-demand inhibition of SAL1 by the synthetic inhibitor V20 establishes a proof of concept for selective manipulation of oxidative stress signaling and sulfur metabolism in Arabidopsis (Khin *et al*., 2026), defining the SAL1–PAP axis as a novel target for the biotechnological improvement of stress resilience of plants.

## Perspectives

We have begun to understand how sulfur supply is integrated into nutrient homeostasis and how general sensor kinases, such as TOR, perceive signals that are attributed to changes in sulfur availability. However, the direct sensing mechanism for external sulfur availability remains unclear and may differ from that for internal sulfur demand. The CSCs in the different subcellular compartments are good candidates for the sensing of intracellular sulfide, but whether the intracellular sulfide concentration reflects the external sulfur supply is unclear. Besides the identification of sulfur sensors, the mechanism of SLIM1 action is also not sufficiently understood, as neither its transcript level, protein abundance, nor localization differ in sulfur-starved and replete Arabidopsis plants. Sulfur sensing and signaling thus will remain an active field of plant sulfur research.

Beyond the transcriptional regulation, several recent reports, including two in this Special Issue, point to an intriguing connection of sulfur metabolism with epigenetics: on the one hand, modulation of sulfur homeostasis, primarily associated with the metabolism of methionine, affects DNA methylation; on the other hand, response to sulfur starvation is at least partly controlled at the epigenetic level. The recent discovery of histone sulfation in mammals (Yu *et al.*, 2023) gives additional impetus to increase the mechanistic understanding of epigenetic processes connected to sulfur metabolism and homeostasis.

Sulfur-derived signals have been implicated in responses to salinity, heavy metal toxicity, pathogen attack, nutrient deficiency, and oxidative stress, highlighting the broad influence of sulfur metabolism on plant adaptation. Furthermore, like sulfur defense compounds, signals derived from sulfur metabolism are coordinated for successful responses to a diverse set of abiotic and biotic stresses. One such example is the coordination of long-distance organ-to-organ communication controlling sulfate distribution, guard cell-specific cysteine production by the pCSC, and protein persulfidation by sulfide at the cellular level, described in the drought stress response. Understanding the impact of sulfur metabolism on responses to abiotic (high light, salinity, heavy metals, and nutrient deficiency) and biotic (herbivory, pathogen attack, and microbial interactions) factors, and integration of the signaling networks are becoming another major thematic area in this field.

Beyond the molecular mechanisms, emerging technologies such as single-cell transcriptomics, spatial metabolomics, and proteome-wide mapping of post-translational modifications are revolutionizing our understanding of plant cell physiology by providing unprecedented resolution of cellular heterogeneity and regulatory dynamics. The application of these novel approaches will enable the dissection of the complex signaling networks across molecular, spatial, and temporal scales that govern cellular responses and plant development under sulfur-limited conditions. These technologies will also be critical for understanding the downstream mechanism of sulfur-dependent signals at the whole-plant level.

**Fig. 1.**
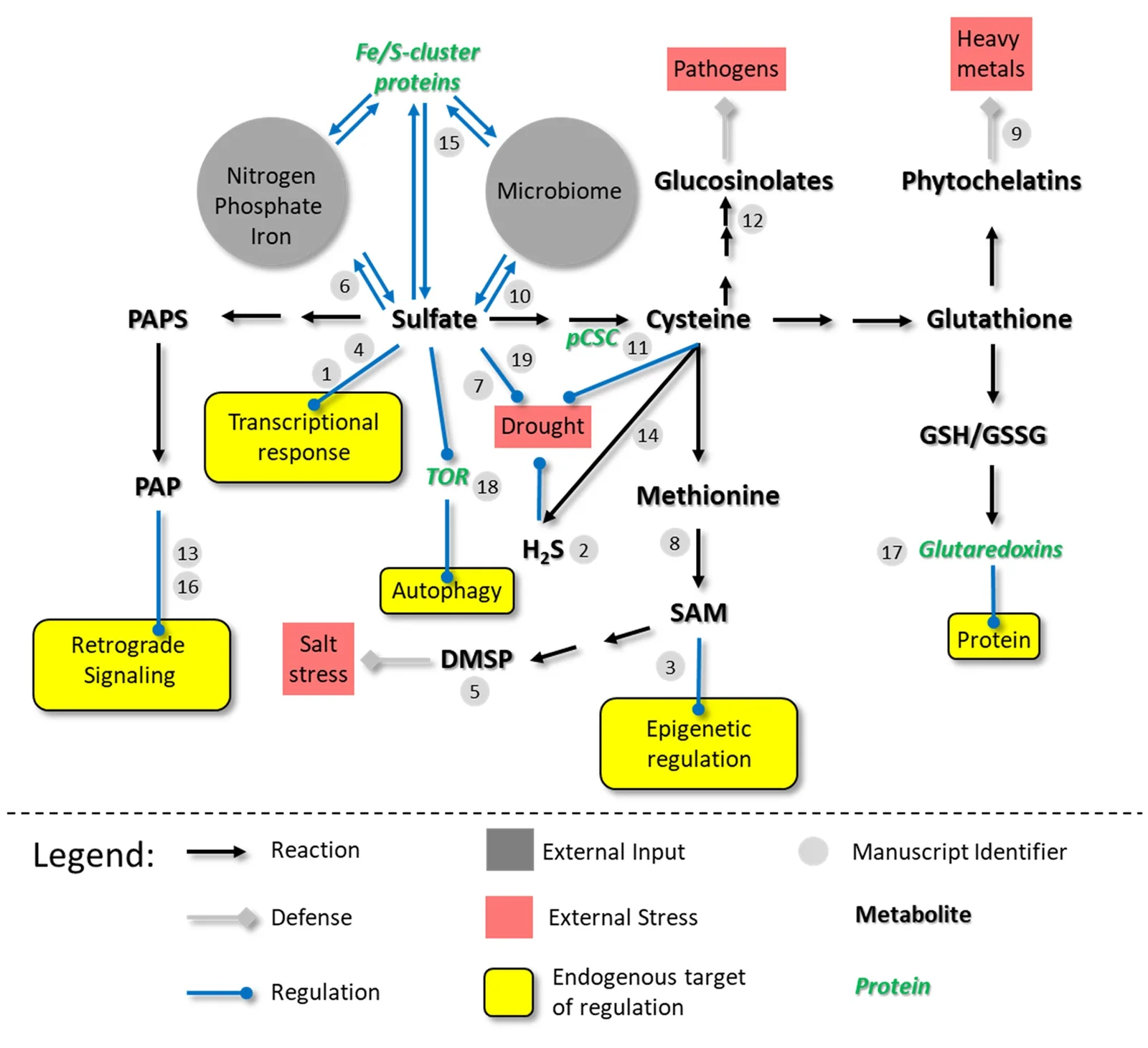
Schematic overview of primary and secondary sulfur metabolism, highlighting key metabolites that act as either signals (blue circles) or defense metabolites (gray diamonds) upon environmental stimuli (dark gray circles) or stresses (red boxes). Sulfur metabolism-derived signals can either control specific response pathways (yellow boxes) or indicate environmental stress, which is regulated by those signals. Invited reviews and articles addressing the diverse interactions between the environment and the sulfur metabolism are indicated by numbers in gray circles. 1, Maruyama *et al*. (2026); 2, Aroca *et al*. (2026); 3, Tremblay *et al*. (2026); 4, Sirko *et al*. (2026); 5, Payet *et al*. (2026); 6, Haque *et al*. (2026); 7, Lehr *et al*. (2026); 8, Amir *et al*. (2026); 9, Wang and Zhao, 2026, 10, Elkatmis *et al*. (2026); 11, Sun *et al*. (2026); 12, Wajn *et al*. (2026); 13, Khin *et al*. (2026); 14, Moseler *et al*. (2026a); 15, Garcia-Godos *et al*. (2026); 16, Furbank *et al*. (2026); 17, Moseler *et al*. (2026b); 18, Vanbuel *et al*. (2026); 19, Bonnot *et al*. (2026).
